# Supplementary material for: Analysis of the Presence and Levels of IgG Antibodies Directed against the S1 Protein Receptor Binding Domain and the N Protein of SARS-CoV-2 in Patients with Multiple Sclerosis Treated with Immunomodulatory Therapies
Source: Vaccines (Basel). 2024 Feb 29;12(3):255. doi: 10.3390/vaccines12030255 (PMC10974963; doi:10.3390/vaccines12030255)
Supplement: Supplementary file 1 [file vaccines-12-00255-s001.zip › vaccines-2856511-supplementary.pdf]

Suplemmentary materials

Table S1. IgG-S1RBD and IgG-N levels according to particular DMTs.

			VISIT I[1]	VISIT II[1]
**INF[2]**	Wholegroup n=14	IgG-S1(RBD)[5]	4645,7 (197,8; 16898)	4878,5 (1061,42; 15088,35)
		IgG-N[6]	0,23 (0,0325; 0,88)	1,035 (0,31; 3,64)
	Vaccinated visit 1 n=7 visit 2 n= 9	IgG-S1(RBD)	16898 (10560,05 ;24896,85)	15264,3 (6997,3; 80000)
		IgG-N	0,05 (0,045; 1,85)	1,54 (1,37; 8,38)
	Unvaccinated visit 1 n=7 visit 2 n=5	IgG-S1(RBD)	197,8 (0,8; 728,5)	1919,5 (79,3; 5581,5)
		IgG-N	0,15 (0,08; 0,83)	0,75 (0,675; 0,78)
**GA[3]**	Wholegroup n=10	IgG-S1(RBD)	8708,55 (1489,925;16946,325)	4659,35 (983,425; 13982,25)
		IgG-N	0,365 (0,04; 1,16)	1,265 (0,2575; 4,03)
	Vaccinated n=8	IgG-S1(RBD)	12351,55 (5966,4; 24059,68)	7428,3 (1073,475; 15030,8)
		IgG-N	0,215 (0,0375; 0,84)	1,625 (0,4525; 5,63)
	Unvaccinated n=2	IgG-S1(RBD)	438<br>4645,7	6709,6<br>359
		IgG-N	0,35<br>1,87	1,48<br>0,16
**DMF[4]**	Wholegroup n=14	IgG-S1(RBD)	3013,8 (12,1; 18568,7)	3886,9 (1162,2; 4165,2)
		IgG-N	0,22 (0,033; 0,522)	0,75 (0,29; 1,99)

Vaccinated (visit 1 n=8 Visit 2 n=10)	IgG-S1(RBD)	14328,25 (5684,7;21981,85)	3954,4 (1358,25; 6942,93)
	IgG-N	0,44 (0,19; 0,61)	0,76 (0,22; 1,95)
Unvaccinated (Visit 1 n=6 Visit 2 n=4)	IgG-S1(RBD)	55,8 (11,73; 220,25)	1148,4 (735,65; 2048,175)
	IgG-N	0,04 (0,015; 0,16)	0,39 (0,29; 1,64)

[1] Data in table are presented as Median (Q1; Q3); [2]Interfero [3] Glatiramer acetale; [4] Dimethyl fumarte; [5]Antibodies IgG against S1protein binding receptor domain;[6]AntibodiesIgGagainst N protein.
